# PA28γ: New Insights on an Ancient Proteasome Activator

**DOI:** 10.3390/biom11020228

**Published:** 2021-02-05

**Authors:** Paolo Cascio

**Affiliations:** Department of Veterinary Sciences, University of Turin, Largo P. Braccini 2, 10095 Grugliasco, Italy; paolo.cascio@unito.it

**Keywords:** PA28γ, PA28αβ, proteasome, proteasome activator, proteasome gate, protein degradation, ATP-independent proteolysis, ubiquitin–proteasome system (UPS), intrinsically disordered proteins (IDPs), proteostasis

## Abstract

PA28 (also known as 11S, REG or PSME) is a family of proteasome regulators whose members are widely present in many of the eukaryotic supergroups. In jawed vertebrates they are represented by three paralogs, PA28α, PA28β, and PA28γ, which assemble as heptameric hetero (PA28αβ) or homo (PA28γ) rings on one or both extremities of the 20S proteasome cylindrical structure. While they share high sequence and structural similarities, the three isoforms significantly differ in terms of their biochemical and biological properties. In fact, PA28α and PA28β seem to have appeared more recently and to have evolved very rapidly to perform new functions that are specifically aimed at optimizing the process of MHC class I antigen presentation. In line with this, PA28αβ favors release of peptide products by proteasomes and is particularly suited to support adaptive immune responses without, however, affecting hydrolysis rates of protein substrates. On the contrary, PA28γ seems to be a slow-evolving gene that is most similar to the common ancestor of the PA28 activators family, and very likely retains its original functions. Notably, PA28γ has a prevalent nuclear localization and is involved in the regulation of several essential cellular processes including cell growth and proliferation, apoptosis, chromatin structure and organization, and response to DNA damage. In striking contrast with the activity of PA28αβ, most of these diverse biological functions of PA28γ seem to depend on its ability to markedly enhance degradation rates of regulatory protein by 20S proteasome. The present review will focus on the molecular mechanisms and biochemical properties of PA28γ, which are likely to account for its various and complex biological functions and highlight the common features with the PA28αβ paralog.

## 1. The Ubiquitin–Proteasome System (UPS)

**Figure 1:**
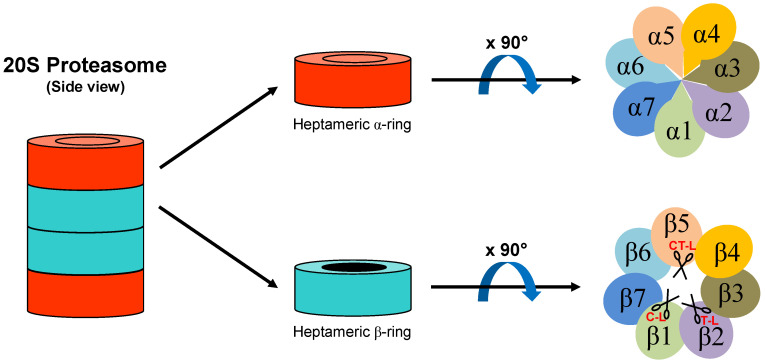
Schematic structure of 20S proteasome. Proteolytic active sites of β1, β2, and β5 subunits are depicted as scissors. C-L: caspase-like; T-L: trypsin-like; CT-L: chymotrypsin-like.

In living organisms, proteins are constantly subject to synthesis and degradation processes that are aimed at rapidly adapting the proteome of the cell to any change in its metabolic and physiological needs, and to guarantee the maintenance of cellular homeostasis challenged by exogenous and endogenous stimuli and stresses. In particular, each cell needs to remove proteins whose function is no longer required at a specific time (e.g., regulatory proteins or transcription factors), damaged proteins, covalently modified (e.g., oxidized) or presenting any other alteration or modification that could be potentially dangerous or toxic, propeptides from inactive precursors, and proteins from which to obtain amino acids to be used as an energy source in case of insufficient caloric intake [1]. In eukaryotic cells, the large majority of intracellular proteins are hydrolyzed by the 26S proteasome, a large (2.4 MDa) multimeric protease abundantly expressed in the nucleus and cytosol [2]. The central core of the 26S proteasome consists of the relatively latent 20S proteolytic particle, at the two free ends of which may associate various regulatory complexes that perform the task of controlling and modulating the functions of the protease in different ways [3]. The 20S proteasome, whose internal cavity harbors proteolytic sites, is the central core of this proteolytic macromolecular machine: it has a cylindrical structure with a molecular weight of ~700 kDa and is formed by four overlapping rings of seven subunits each. The two outer rings are constituted of α subunits, and the two inner ones of β subunits [4]. The active catalytic sites of the constitutive 20S proteasomes are located on the β1, β2, and β5 subunits (Figure 1). In principle, proteasomes can hydrolyze the C-terminal amide bond of any amino acid except proline. However, proteolytic activities assessed by short fluorogenic peptides have identified three well-defined hydrolyzing preferences: trypsin-like (i.e., hydrolysis at the C-terminus of basic residues, performed by β2 subunit), chymotrypsin-like (i.e., hydrolysis at the C-terminus of hydrophobic residues, performed by β5 subunit), and caspase-like (i.e., hydrolysis at the C-terminus of acidic residues, performed by β1 subunit) [5].

Under the stimulus of γ-interferon (INF-γ) or other pro-inflammatory cytokines, new catalytic subunits are synthesized (β1i, β2i, and β5i) that replace the constitutive one to form newly assembled 20S immunoproteasomes [6]. Experiments with small fluorogenic peptides demonstrated that immunoproteasomes possess a greater ability to hydrolyze after hydrophobic amino acids and a reduced capacity to cleave after acidic residues, while conflicting results are reported concerning its capacity to cleave after basic residues [7,8]. Consequently, peptides produced by immunoproteasomes are expected to have a greater amount of hydrophobic and a lower amount of negatively charged C-termini, which favors uptake by TAP transporters and tight binding to MHC class I molecules [9]. Crystal structures of the mouse constitutive and immuno 20S proteasomes revealed differences in the substrate-specificity pockets between the catalytic constitutive and immuno β subunits, which largely provide an explanation for the observed differences in cleavage specificities [10]. In fact, whereas the substrate pocket of β1 accommodates an acidic P1 residue, that of β1i interacts with a small, hydrophobic P1 residue. Moreover, the active sites of both β5 and β5i are surrounded by nonpolar environments, but the pocket of β5i is significantly larger than that of β5, thus enabling accommodation of a bulky, hydrophobic P1 residue. Finally, the substrate-binding pockets of the mouse β2 and β2i subunits are essentially identical, which appears in contradiction with the enhanced trypsin-like activity of immuno vs. constitutive proteasomes reported in several studies [11,12,13]. However, biochemical kinetic analyses have often been performed with human or rabbit proteasomes, and as such species-specific structural differences could possibly account for this discrepancy. In this regard, it is worth noting that, unlike in humans, mouse MHC class I molecules never accommodate peptides with basic residues at their C-terminus [9]. Although it has also been reported that some epitopes, especially self-antigens, are poorly generated by immunoproteasomes [14], the pivotal role of immunoproteasomes in the generation of the vast majority of MHC class I ligands was definitively demonstrated in transgenic mice lacking all three proteasomal catalytic β-immune subunits [15]. Moreover, immunoproteasomes have been shown to be important for efficient cytokine production [9] and have been implicated in a number of pathological disorders such as cancer and neurodegenerative and autoimmune diseases [16]. Finally, it has been demonstrated that rabbit 26S immunoproteasomes possess the capacity to hydrolyze basic proteins (such as histones and myelin basic protein) at greatly increased rates compared to constitutive proteasomes [17].

**Figure 2:**
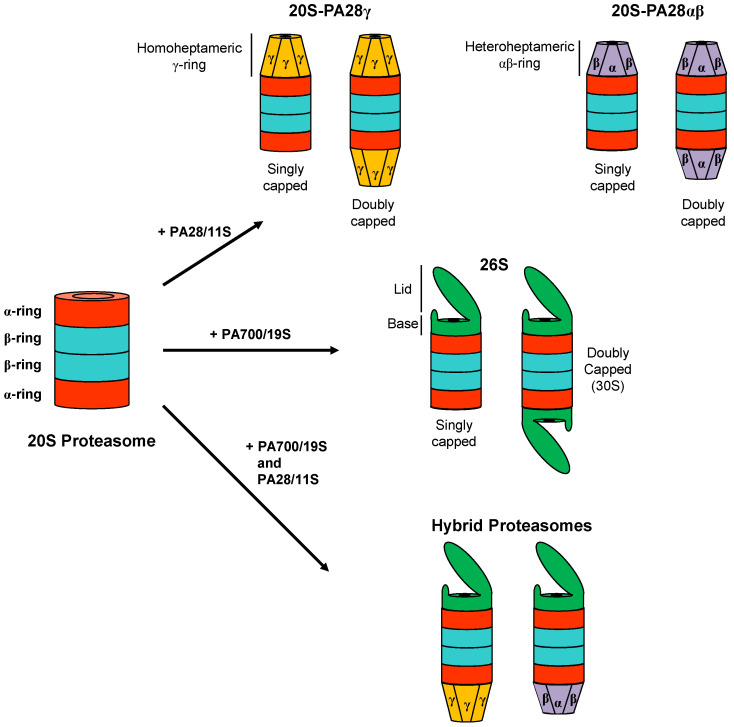
Different proteasome forms present in mammalian cells. Schematic structures of 20S, 26S, 20S-PA28αβ, 20S-PA28γ, and hybrid proteasomes are displayed.

One of the complexes that activate the 20S proteasome, whose functions and mechanisms of action are better understood, is the 19S regulatory particle [2] that, once associated with one or both ends of the 20S, leads to the formation of the 26S proteasome [1] (Figure 2). The 19S regulatory cap performs the task of recognizing, unfolding, and trans-locating protein substrates within the protease [18]. Only proteins without tightly folded three-dimensional structures can, in fact, pass through the narrow aqueous pore present in the center of the α-ring of the 20S particle [19]. Moreover, to further limit uncontrolled access of substrates inside the inner proteolytic chamber, the pore of the α-annulus is blocked by a gate consisting of the intertwining N-terminal ends of the proteasomal α-subunits [20,21]. The 19S regulatory particle is composed of two subcomplexes, namely the lid and the base [1]. The lid is formed by the association of nine non-ATPase subunits (Rpn3, Rpn5–Rpn9, Rpn11, Rpn12, and Rpn15/Sem1) that fulfil important scaffolding functions. Moreover, Rpn11 is Zn^++^-dependent protease that removes poly-ubiquitin chains from substrates to be degraded [2]. The base is formed by three ubiquitin receptors (Rpn1, Rpn10, and Rpn13), Rpn2, and a ring of six different AAA+ family ATPases (Rpt1–Rpt6) that form a central channel [22]. The Rpt1–6 heterohexameric ring constitutes the molecular motor of the 26S proteasome that uses ATP-dependent motions to unfold and translocate protein substrates into the 20S central proteolytic particle [23]. To do this, Rpts use conserved loops projecting from AAA+ domains into the central pore of the motor to physically engage disordered regions within the protein substrate, applying a mechanical pulling force generated from repeated cycles of ATP hydrolysis for unfolding, and then transferring the unfolded polypeptide thorough the translocation channel into the 20S core [24,25]. Moreover, the AAA+ ATPases open the gate formed by the N-termini of α-subunits that seals the entrance pore of latent 20S particle [25,26,27]. Specifically, five of the ATPases (Rpt1–3 and Rpt5–6) present a hydrophobic-tyrosine-X (HbYX) or related motifs at their C-terminus that insert into the 20S inter α-subunit pockets to induce gate opening [25,28,29]. Importantly, the same pockets are also utilized by PA28 activators to associate with the 20S core particle, but in this case opening of the gate occurs by a different molecular mechanism which is described below.

The affinity of the native folded proteins for the 26S proteasome is, however, extremely low, and this is why it is necessary to increase it considerably through the binding of the protein, which must be hydrolyzed, with a polyubiquitin chain. This chain is synthesized by three classes of enzymes (E1, E2, and E3) that selectively recognize a target protein and modify it through the subsequent addition of multiple molecules of the small protein ubiquitin, linked to the substrate to be hydrolyzed, and to each other, through isopeptide bonds [30]. The 19S particle harbors, as previously mentioned, specific receptors for the resulting polyubiquitin chain, the presence of which, together with an unstructured segment (generally at the C- or N-terminal end, but which may also be present in the internal sequence of the substrate), from which the unfolding and subsequent hydrolysis can begin, constitute the two fundamental signals required for the degradation of a protein by the 26S proteasome [23].

## 2. The PA28 Family of 20S Proteasome Regulators

### 2.1. Structure of PA28s

**Figure 3:**
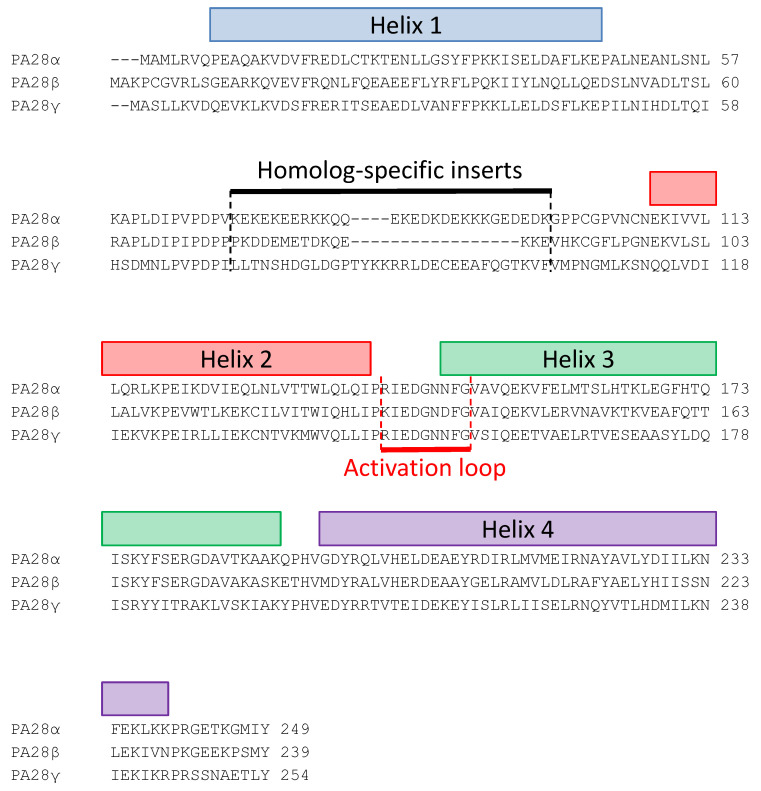
Sequence alignment of the three human PA28 subunits. Secondary structure, homolog-specific inserts, and activation loop are displayed.

**Figure 4:**
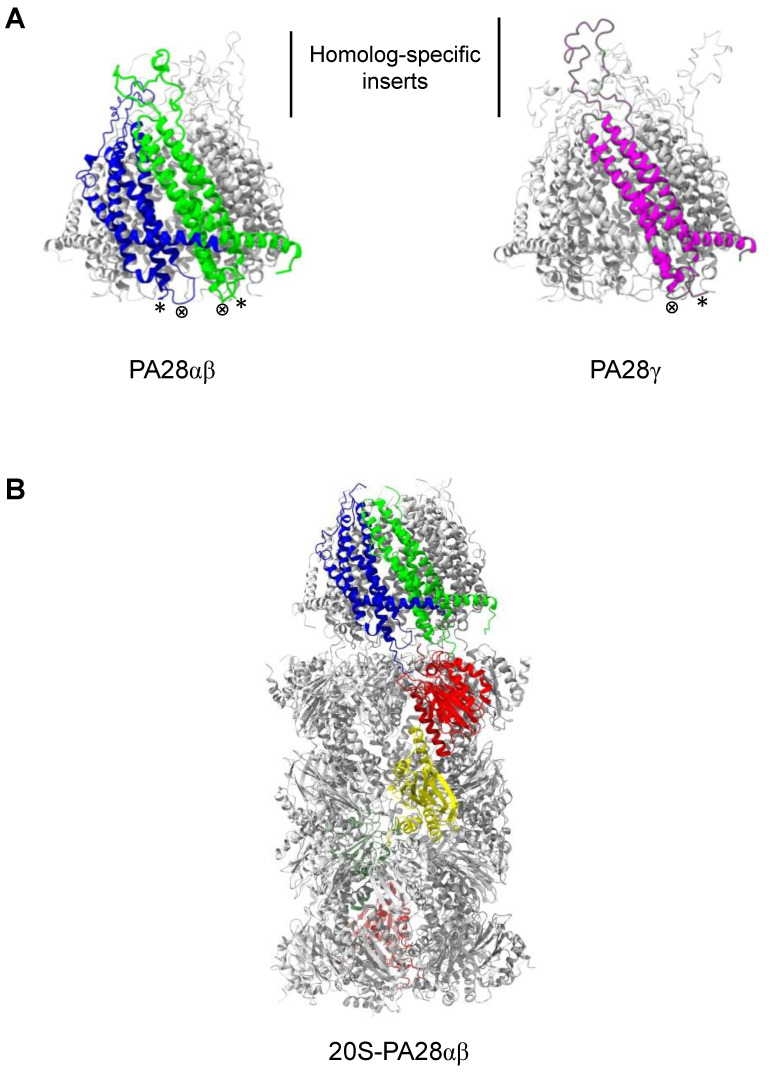
Model structure of PA28αβ, PA28γ and 20S-PA28αβ complexes. (**A**) Structure of the heteroheptameric PA28αβ (left) and homoheptameric PA28γ (right). One α (blue), one β (green) and one γ (magenta) subunit are highlighted. * indicates C-terminus; ⊗ shows activation loop. Structures were generated by ChimeraX using the PA28αβ crystal structure [45]. (**B**) Structure of 20S-PA28αβ proteasome. One α (blue) and one β (green) subunit of PA28 and α1 (red) and β1 (yellow) subunits of 20S are highlighted. Structures were generated by ChimeraX using the 20S-PA28αβ EM structure [51].

Another important family of 20S proteasome regulators is that of the ATP- and ubiquitin-independent PA28 activators (also called 11S, REG or PMSE), which in vertebrates is formed by the three highly homologous subunits α, β, and γ [31]. PA28 monomers in vivo and in vitro form ring-shaped ~200 kDa multimeric complexes that bind, in an ATP-independent manner, to the two ends of the 20S proteasome and profoundly modifies its peptidase, and in the case of PA28γ also its proteolytic, activities [31]. In addition, PA28 rings can also associate with the free end of asymmetric 26S proteasomes (19S-20S) to form so-called “hybrid proteasomes” (19S-20S-PA28) [32,33,34,35,36] (Figure 2) that hydrolyze tri- and tetra-peptides at higher rates than canonical 26S particles and generate a different spectrum of peptide products from full length substrate proteins [32,34,37]. PA28 proteins have an apparent molecular weight of 28 kDa on SDS-PAGE electrophoresis and share ~50% (PA28α vs. PA28β), 40% (PA28γ vs. PA28α) and ~32% (PA28γ vs. PA28β) amino acid sequence identity [38,39]. Based on the crystal structure of the PA28α homoheptameric ring solved by Hill et al. [40], the overall secondary structure of PA28 monomers is composed of four long α-helices of 33–45 residues in length that are involved in intra- and intermolecular interactions. The tertiary structure of the PA28 monomer is mainly stabilized by hydrophobic interactions between helices and a few buried polar associations. The linker sequence between helices 2 and 3, which is highly conserved in all three PA28 subunits, is designated the “activation loop” since it is responsible for stimulation of proteasome peptidase activities, as shown by mutagenesis studies on this region [41]. A second part of the molecule involved in the interaction with the 20S particle is the 10 residue C-terminal tail that provides binding energy for PA28-proteasome association without, however, directly stimulating 20S enzymatic activities [42]. Finally, the linker between helices 1 and 2 is composed of sequences that are highly divergent between the three PA28 subunits and for this reason are known as “homolog specific inserts” [43]. Although these inserts are not resolved in the X-ray structure of PA28α, presumably since they are flexible and it is almost certain that they protrude from the upper surface of the PA28 rings [31] (Figure 3). Interestingly, the insert of PA28γ is longer than those present in the PA28α and PA28β subunits, which could at least partially account for the marked differences in the biochemical properties of the homoheptameric compared to heteroheptameric PA28αβ (see below). Although it is well established that in vitro recombinant PA28α can form a homoheptameric ring [40,44], recombinant PA28β has been reported to be both a monomer [41,43] and a homoheptamer [45,46]. The subunit stoichiometry of native heteroheptameric PA28αβ is still under debate, with some studies claiming that it is formed by the association of three α and four β subunits [47,48] and others indicating four α and three β subunits [45]. In contrast, there is no doubt about the quaternary structure of native PA28γ, which is definitely a homoheptameric ring [49,50] (Figure 4A). It is also well-established that the PA28 homo and heteroheptameric rings assemble and are stabilized thanks to both polar and hydrophobic interactions [40]. Specifically, helix 2 of one monomer associates with helix 4 of the adjacent subunit. This quaternary donut-like structure is further stabilized by interactions between the N-terminal portions of helices 1 that project away from each monomer and interact with helices 1 and 4 of the neighboring molecules, thus forming a kind of belt around the outside of the heptamer. Electron microscopy images show that PA28αβ forms a cap on the end of 20S particle, which is about 10–11 nm wide at the base where it attaches to proteasome outer α ring and 7–8 nm in length from the base to the tip [51,52] (Figure 4B). Electron microscopy data also suggest that PA28αβ contains a central channel, which apparently traverses it entirely to the central pore of proteasome α-annulus. Accordingly, the crystal structure of recombinant PA28α at 2.8 Å resolution reveals a heptameric ring traversed by a central aqueous channel with a diameter of 30 Å on the face contacting the proteasome and 20 Å on the other [40]. This structural organization of PA28 rings has been recently confirmed by a crystallographic study of PA28αβ heterocomplexes [45]. Of great interest, the inner surface of PA28 ring is almost completely lined with charged or polar residues from helices 3 [40], which are likely to impose a strong constraint on the bidirectional transit of peptides through the aqueous channel; this gives rise to, at least in part, the biochemical properties of PA28 activators [37,53]. An similar quaternary organization has also been reported for a PA28 homolog present in *Plasmodium falciparum* (PfPA28), whose structure in association with the homospecific 20S particle was recently published [54]. In this case, PfPA28 was found to bind 20S proteasome asymmetrically, strongly engaging subunits on only one side of the core particle. Interestingly, the inner channel of the homoheptameric bell-shaped PfPA28 particle, which connects the internal proteolytic cavity with the external medium, presents a strong segregation of charges, in line with what has been observed for PA28s in vertebrates.

### 2.2. Opening of the Proteasomal Gate

Despite considerable efforts over the past 30 years, a complete and definitive understanding of the molecular mechanisms underlying the regulation of 20S enzymatic activities by PA28s is still missing. In this regard, the most important acquisition, obtained 20 years ago, was the demonstration that PA26 (the PA28 homologue in trypanosomes) opens the gate that normally seals the pore in the center of the proteasomal α-ring, through which the substrates enter and products emerge from the proteolytic cavity of the 20S core particle [55]. This discovery certainly represented a fundamental point in studies on PA28s, although it is now clear that by itself is unable to explain all the biochemical properties and activities described for this activator. In the crystal structure solved by Hill and coworkers, PA26 C-terminal residues were shown to dock into pockets between adjacent proteasome α subunits and, by forming hydrogen bonds and a salt bridge between the C-terminal carboxylate of the activator and a highly conserved proteasome lysine side chain (Lys 66), provide binding energy for PA26-20S complexes [55,56]. Binding to the C-termini of PA26, however, is not sufficient to activate the 20S proteasome, which requires the activation loop that forms a 7-fold symmetric circular array that interacts with the base of the N-terminal gating residues of the seven proteasomal α subunits. In particular, a glutamate side chain (Glu 102) in each PA26 subunit activation loop contacts and repositions a proline residue (Pro 17) of 20S α subunits located above the surface of the proteasome. This interaction triggers gate opening by disrupting packing and hydrogen bonding interactions of the asymmetrical closed conformation and by widening the pore opening to a more symmetrical arrangement that allows a belt of intersubunit contacts to form around the circumference of the opening [42,57]. Importantly, the open conformation is stabilized by interactions within a cluster of four conserved proteasomal residues (Tyr 8, Asp 9, Pro 17, and Tyr 26 from each α subunit) that pack against each other at the interface between each of the adjacent α-subunits [42,56,57]. Therefore, repositioning of Pro 17 induced by PA26 activation loops not only destabilizes the closed conformation, but also allows formation of stabilizing interactions between Tyr 8/Pro 17 of the subunits and Asp 9/Tyr 26 of the contiguous one. Of note, these four residues are highly conserved (from archaea to human), even in species that do not express PA28 activators [42]. This observation, therefore, suggests that the same open gate proteasomal conformation may also be induced by the ATP-dependent activators, which are found in all species that express proteasomes. Accordingly, in vitro degradation of a model protein substrate by archaeal 20S proteasome in association with the ATPase activator PAN was strongly reduced following mutation of Tyr8, Asp9, Pro17, or Tyr26 [57].

A related but distinct mechanism for opening the proteasomal gate has also been described for PfPA28 [54]. In this case, PfPA28 was shown to destabilize the N-termini of the α2, α3, and α4 subunits, which in the uncapped *Plasmodium* proteasome block the pore, thereby allowing access into the internal proteolytic chamber, but in this case the activation loops interact exclusively with the N-termini of the α1, α5, α6, and α7 proteasomal subunits.

### 2.3. More than Opening of the Gate

Although it is now widely accepted that members of the PA28 family act through the opening of the proteasomal gate, it is also clear that this molecular mechanism alone is not able to explain all the biochemical effects described over the years on the enzymatic activities of the 20S [53]. In fact, the activation kinetics of PA28s vary greatly between different fluorogenic peptides (e.g., in terms of extent of stimulation) and, importantly, also differ in term of the peptides hydrolyzed by the same proteasomal catalytic site, which cannot be explained by the mere widening of proteasomal α-pore [43,58,59,60]. Furthermore, mean and median sizes of peptide products released during degradation of full-length proteins by 20S proteasomes are significantly decreased in the presence of PA28αβ [37], but unaffected in the presence of PA28γ [61]. Crucially, this observation further argues against a simple dilation of the pore of 20S α-annulus induced by PA28s, since in this case a prevalent release of longer peptides (which can more easily diffuse outside of the proteolytic chamber, thus avoiding further fragmentation) should be observed, in line with data obtained with a constitutively open gate proteasome mutant [27]. Therefore, it appears very likely that PA28s also exert their stimulatory activity through other mechanisms in addition to the opening of the gate. Among all those proposed during the years, the allosteric modification of 20S particle active sites [31,38] and/or action of a selective molecular sieve appear to be the most probable [53].

### 2.4. Allosteric Modifications of Active Sites

PA28 activators have frequently been proposed to enhance hydrolysis of short peptides by inducing long-range conformational changes in proteasomal active sites. Accordingly, it has been reported that 20S catalytic sites are allosterically regulated [5,50,62,63,64,65] and that their modification results in opening of the gate that seals the aqueous pore in the α-ring [66,67,68]. Furthermore, an allosteric pathway connecting PA26 docking pockets present on the outer surface of the α-ring of *Thermoplasma acidophilum* 20S proteasome with the internal hydrolytic active sites of the proteases has been reported [69]. Moreover, a complex network of long-range conformational modifications involving 20S core particle α, β, βi, and PA28s subunits was recently suggested on the basis of an interesting hydrogen-deuterium eXchange coupled to mass spectrometry (HDX-MS) approach [70]. In contrast to these observations, however, structural data clearly indicate that binding of PA26 with the 20S proteasome does not result in any allosteric modification of proteolytic β-subunits [55]. In this regard, however, it remains still possible that slight structural mutations in the catalytic sites of 20S proteasomes induced by PA28 are lost in the rigid crystal structure of PA26-20S [71,72].

### 2.5. A Molecular “Smart” Sieve

A second model, not necessarily an alternative to the previous one, postulates that PA28s can also act as a molecular sieve that hinders the release of larger peptides until they are further fragmented into smaller pieces [53]. This possibility is consistent with detailed kinetic studies demonstrating that PA28αβ achieves its stimulatory activity by increasing bi-directional transit of 3–4 amino acid peptides [73]. Furthermore, careful in vitro/in silico analysis established one of the major functions of PA28αβ is that of decreasing the efflux of long peptide products out of the internal proteolytic 20S chamber [74]. Moreover, it was also shown that a heteroheptameric PA28αβ particle lacking the unstructured and highly mobile PA28α homolog specific inserts, surrounding the central channel of the activator, stimulates hydrolysis of peptides longer than nine residues more strongly than wild type PA28αβ. Consequently, it was suggested that the flexible loops of PA28α might act as gatekeepers that hamper the exit of longer peptides from the 20S proteolytic particle [47]. Selectivity based exclusively on peptide size, however, cannot explain all the effects of PA28αβ described by Raule et al. [37]. In fact, quantitation of all products released following proteasomal degradation of full-length proteins, showed that several individual long peptides (8–23 residues in length) are released in much higher amounts by PA28αβ-20S than by free 20S. Therefore, PA28αβ is likely to act as a selective “smart” filter that allows facilitated efflux of a subset of individual longer products. This specific selection is likely to be dictated by the large number of charged or polar amino acids that almost completely cover the surface of the central channel of the PA28 heptameric rings.

## 3. PA28γ

### 3.1. General Properties

While PA28α and β are both highly up-regulated by γ-interferon and their main biological function is related to MHC-class I antigen presentation [53], PA28γ (also known as REGγ, 11Sγ, PSME3, or Ki antigen) has a predominantly nuclear localization that is not significantly induced by interferon-γ and whose biochemical and cellular properties appear to be significantly different from those of the other two members of this family of proteasome activators [75]. PA28γ was initially described as a major autoantigen of patients with systemic lupus erythematosus [76], although its direct link with the autoimmune syndrome requires further clarification. Subsequent studies later revealed that this protein shares a high degree of homology with PA28α and β and represented a genuine activator of the 20S proteasome [43,77,78]. However, a more recent label-free quantitative proteomic analysis estimated that less than 5% of the total amount of PA28γ in the cell is associated with the 20S particle [79]. This observation suggests that some of its functions might also be proteasome-independent or, alternatively, that this large fraction of free PA28γ represents a dynamic stock that is ready to be assembled with proteasomes in response to specific cellular needs or conditions [80]. Recent phylogenetic analyses showed that PA28γ is widely present in both jawless and jawed vertebrates, while expression of PA28α and β is restricted to only jawed ones, with the notable exception of birds [81]. The most likely scenario emerging from these studies is that PA28γ represents a slow-evolving gene from which faster evolving sequences stemmed out, leading to PA28α and β. In line with this hypothesis, the most conserved paralog (i.e., PA28γ) is likely to have retained the ancestral function of the PA28 precursor. On the contrary, the more recent ones (PA28α and β), which originate from a copy that diverged at a high rate soon after the first duplication event, have acquired new properties in line with their role in adaptive immune responses [81,82]. Accordingly, in mammals PA28αβ is constitutively expressed only in lymphoid cells and immunological organs, but its levels rise dramatically in virtually any other tissue in response to interferon-γ or other pro-inflammatory cytokines (e.g., TNF-α or interferon-α and -β) [31,53], concomitantly with the levels of other components of the MHC class I antigen presentation pathway [8,83]. On the contrary, PA28γ is widely distributed in every organ of the body [77,84,85,86], with apparently high and specific expression in the spleen and brain [85]. Notably, discordant results have been described in the literature regarding regulation of PA28γ by interferon-γ. In fact, interferon-γ was reported to modestly and only transiently increase levels of PA28γ mRNA [84], [87], to have no effects on mRNA levels but strongly reduce protein expression [77], and to reduce protein expression in an antiviral immunoresponse in mouse liver, but not in several cell lines [88] (Table 1). The general picture that emerges from these studies is that PA28γ is only marginally, if at all, affected by interferon-γ, in agreement with the observation that the promoter region of its gene does not contain sequence motifs that are identifiable as interferon-stimulated response elements [89]. Furthermore, no change in PA28γ mRNA levels could be detected after heat shock and innate immune induction [82], while strong up-regulation was reported in response to serum stimulation in murine embryonic fibroblasts [90]. Moreover, alterations of its cellular abundance are well documented in some diseases (especially in several tumors) [75,91], pointing out a possible role in these pathological conditions, as will be discussed below. PA28γ homoheptamers and PA28αβ heteroheptamers also differ in terms of their subcellular localization. While PA28αβ is distributed in both the nucleus and cytosol [31], PA28γ is primarily localized in the nucleus but absent from the nucleolus, although some cytosolic expression has also been reported [82,92,93,94,95,96]. Accordingly, the sequence of PA28γ has two functional nuclear localization signals (NLS), one present in the homolog-specific insert region and the second at the N-terminus [82,89], which account for its preferential presence in the nuclear compartment [92].

### 3.2. Biological Functions

#### 3.2.1. Role in Cell Growth and Proliferation

Although a role of PA28γ in regulating cell growth was suggested more than 30 years ago [90], only with the generation of PA28γ^−/−^ transgenic mice has it been possible to better clarify its function in controlling the cell cycle [97,98]. Deletion of the PA28γ gene did not result in mortality during embryogenesis or fetal development and transgenic mice were born without obvious abnormalities in any tissues or organs examined; moreover knockout mice were fully viable and fertile. However, PA28γ-deficient mice grew more slowly after birth and reached a smaller body size compared to WT or heterozygous littermate controls. Importantly, this reduced body size seemed to be the consequence of a retardation of cell proliferation. In fact, PA28γ^−/−^ MEFs were characterized by a reduced number of actively mitotic cells in both the S and G_2_/M phases as a consequence of an evident slowdown of the transition from G_1_ to S phase of the cell cycle [97,98]. This role of PA28γ in controlling cell cycle progression and cellular proliferation was subsequently also demonstrated in Drosophila [94]. In this organism, a sequence search of the PA28γ promoter region typically identified transcription regulatory elements (DNA replication-related elements, DRE) in genes involved in cell cycle progression and DNA replication. In line with this finding, silencing of the PA28γ gene thorough RNA interference resulted in partial arrest in the G1 phase of Drosophila cells, thus confirming its role in cell cycle regulation in invertebrates. Many studies subsequently confirmed this function of PA28γ in promoting cellular growth and progression thorough the cell cycle in various human cell lines [99,100,101,102,103,104], although an opposite effect has also been reported [105].

#### 3.2.2. Role in Apoptosis

Multiple studies have highlighted a role of PA28γ in regulating apoptosis. Initial observations showed that murine embryonic fibroblasts (MEFs) from PA28γ knockout mice present significantly higher levels of apoptosis than WT controls [97]. A yeast two-hybrid screening of an adult brain cDNA library identified PA28γ as a substrate of caspases 3 and 7, and its susceptibility to in vitro and in vivo cleavage by the two proteases was also documented [106]. Moreover, it has been also shown that increased cellular levels of PA28γ lead to inhibition of caspase activity thorough an unknown mechanism that likely involves ubiquitination and subsequent proteasomal degradation. Accordingly, it was suggested that PA28γ and effector caspases might mutually restrict each other in through a negative feedback loop [107]. In line with this, several studies established a direct correlation between reduced cellular levels of PA28γ and increased susceptibility to apoptosis in many cell types, including various human cancer lines [100,102,103,108], T-cells [109], and spermatogonial mouse cells [110].

#### 3.2.3. Role in Cancer

PA28γ is overexpressed in several types of cancer [111,112,113,114,115,116] and on the basis of this observation its role as an oncoprotein that favors tumorigenesis is widely accepted [91,117]. In fact, its biological functions described in the previous paragraphs, and in particular its stimulating activity on cell growth and proliferation together with its ability to reduce apoptosis, can certainly justify, at least in part, its role in promoting tumor growth. Furthermore, other important processes in which PA28γ is involved, and which can help explain its cancer-promoting effects, concern its additional activities, such as immunosuppressive (see below), pro-angiogenic [118,119], and stimulation of glycolysis [104] and the epithelial-mesenchymal transition (EMT) [109,120,121], thereby aimed to adapt cell metabolism to energy starvation [122].

#### 3.2.4. Role in Lipid Metabolism and Atherosclerosis

A role of PA28γ in regulating lipid metabolism in the liver has also been documented [123]. In fact, mice deficient for this proteasome activator are characterized by a significant enhancement of autophagy in hepatocytes with consequent protection against steatosis induced by high-fat diet. This effect was mediated by PA28γ-induced Ub-independent proteasomal degradation of SirT1, an enzyme that stimulates autophagy by deacetylating Atg5 and 7. Interestingly, a protective effect of low levels of PA28γ towards hepatitis C virus (HCV) core protein-induced steatosis has also been reported [124]. Strictly correlated with its function in lipid metabolism, PA28γ has also been shown to play an important role in preventing formation of atherosclerotic plaques by inhibiting the uptake of modified lipoproteins by macrophages, thus preventing their transformation into cholesterol-laden foam cells [125]. In contrast with this result, however, high expression of PA28γ in atherosclerotic plaques and its role in promoting their formation, through induction of epithelial cells apoptosis, has also been described [126]. The reasons for these conflicting results are presently unclear and emphasize the need to further investigate the role of PA28γ in the etiopathogenesis of atherosclerosis.

#### 3.2.5. Role in Neurodegenerative Diseases

PA28γ has been shown to enhance cell survival in an in vitro model of Huntington’s disease (HD), a neurodegenerative syndrome caused by a mutation that leads to an abnormally long polyglutamine (polyQ) expansion in the huntingtin (Htt) protein [127]. Gene therapy by injection of lenti-PA28γ virus into the striatum of a Huntington’s disease mouse model leads to improvement of motor coordination of the transgenic animals [128]. Furthermore, two opposite effects of PA28γ on motor neuron viability have been described in cell models of spinal and bulbar muscular atrophy (SBMA), a disease caused by expression of a polyglutamine (poly-Q)-expanded androgen receptor (AR) [129]. Depending on the cellular context, PA28γ either exacerbates polyQ-expanded AR toxicity, apparently in a proteasome-binding independent mechanism, or promotes cell viability via a proteasome-binding pathway.

#### 3.2.6. Role in Fertility

PA28γ has been reported to play an indispensable role in male fertility in association with PA200, another ATP- and ubiquitin-independent proteasome regulator. In fact, although PA28γ^−/−^ (and also PA200^−/−^) male mice are fertile, PA28γ/PA200 double knockout (dKO) are completely infertile due to strong reduction in spermatozoa mobility [130]. Interestingly, dKO spermatozoa show increased levels of oxidative damage, which suggests that PA28γ in association with PA200 in these cells might play a specific role in the oxidative stress response. A subsequent study investigating the role of PA28γ in the process of spermatogenesis established that PA28γ knockout male mice are actually subfertile, due to defects in spermatogenesis with a consequent decrease in sperm concentration and reduction of their mobility [110]. Altogether, these data seem to indicate that PA28γ plays an important and specific role in the maturation process of spermatozoa.

#### 3.2.7. Role in Immune Responses

The first studies conducted 20 years ago to evaluate a possible role of PA28γ in regulating the immune response, and in particular MHC class-I antigen presentation, reported negative results [97,98]. In fact, the only immunological defects that could be identified in PA28γ^−/−^ mice were a slight reduction in CD8^+^ cells and impaired clearance of pulmonary fungal infection. Furthermore, surface expression of MHC class I molecules was normal, as was the T cell response to two viral infections both in terms of magnitude and hierarchy of antigenic epitopes recognized [98]. However, more recent studies pointed out that PA28γ has specific functions that are related to the proper functioning of the immune system. Overexpression of PA28γ was shown to markedly decrease MHC class I presentation of epitopes derived from PTPs (Pioneer Translation Products) in cancer cell lines, while its reduction leads to the opposite result [131]. PTPs are polypeptides produced in the nucleus by a non-canonical translation event prior to mRNA splicing and whose generation is specifically enhanced in cancers due to aberrant mRNA transcription and maturation processes that are typically associated with oncogenesis. Therefore, PTPs represent an important source of tumors-associated antigens (TAAs) that are crucial to elicit an effective anti-tumor immune response [132,133,134]. Notably, the PA28γ-induced decrease in PTPs-derived MHC class I antigen presentation strictly depends on the ability of PA28γ to modulate the proteolytic activities of 20S proteasomes, strongly indicating a direct role on the process of epitopes processing, rather than some indirect effect on the abundance or functioning of other components of the MHC class I pathway. Accordingly, in vitro degradation with purified proteasomes of a long precursor (52mer) containing an embedded epitope sequence showed complete hydrolysis into small fragments of the antigenic peptides when 20S was associated with PA28γ, while in its absence generation of the epitope was clearly detectable. Consistent with these observations, knockout of PA28γ was found to negatively impact tumor growth in a mouse model, an effect that was partly dependent on the ability of PA28γ to impair the immunosurveillance process [131]. The ability of PA28γ to reduce MHC class I presentation and to counteract autoimmunity was also recently reported by Yao L. et al. [135]. Interestingly, in this case the immunosuppressive effect observed in mouse bone marrow-derived and splenic dendritic cells (DCs), MEFs, and HeLa cells seems to be achieved through down-regulating the expression of immunoproteasome consequent to enhanced proteasomal degradation of phosphorylated STAT-3. However, in other mouse and human cell lines no major effect of PA28γ on the expression levels of immunoproteasome in basal conditions or under interferon-γ stimulation could be detected [131]. Further studies are thus necessary to establish the role of PA28γ in modulating the levels of immunoproteasome in different cell types and in physiological and pathological contexts. Furthermore, a recent study unveiled a different molecular and cellular mechanism by which PA28γ can attenuate autoimmune reactions, mainly by promoting the degradation of the transcription factor interferon regulatory factor 8 (IRF8) in DCs. In fact, elevated cellular levels of IRF8 resulting from the knockout of the PA28γ gene was found to lead to an increased release of transforming growth factor-β1 (TGF-β1) by DCs. The higher levels of secretion of this cytokine (and also interleukin 6) by DCs causes, in turn, an augmented polarization of interleukin-17A-producing CD4^+^ T cells (Th17), and the onset of an experimental autoimmune encephalomyelitis (EAE) in a mouse model [136]. Moreover, PA28γ was also reported to properly regulate immunoglobulin class switching that initiates antibody gene diversification in activated B lymphocytes by targeting the activation-induced deaminase (AID) for proteasomal degradation [137]. Regarding a possible function of PA28γ in innate immunity and host defense against infections by bacterial pathogens, a positive correlation between PA28γ levels and resistance to infection of *Staphylococcus aureus* [138] and *Listeria monocytogenes* has been reported [139], which in both cases is attributable to regulatory activity on the NF-kB pathway in macrophages.

#### 3.2.8. Role in Chromosomal Stability and Nuclear Dynamics

Several lines of evidences indicate that PA28γ plays an important role in regulating nuclear structure and organization, and intriguingly some of these functions seem to be independent of its capacity to activate proteasomes. PA28γ is localized on chromosomes during interphase and, importantly, its overexpression weakens the mitotic arrest induced by spindle damage, while its depletion has the opposite effect. Furthermore, indicative of its functional role in maintaining centrosome and chromosomal stability, cells depleted of PA28γ are characterized by strong aneuploidy, increased numbers of centrosomes, and multipolar spindles [92]. Moreover, PA28γ-20S complexes were found to be specifically present in nuclear speckles (NS), and depletion of PA28γ triggers a marked modification of NS organization and protein composition [140]. Additionally, PA28γ accumulates in Cajal bodies (CB) following UV-C treatment and its accumulation causes CB fragmentation [141] and appearance of promyelocytic leukemia protein (PML) nuclear bodies (PML-NBs), whose abundance is inversely proportional to the levels of PA28γ [142].

#### 3.2.9. Role in DNA Repair

Specific involvement of PA28γ in the cellular response to DNA double-strand breaks (DSBs) has been reported [143]. In particular, PA28γ depletion was found to enhance cellular sensitivity to radiomimetic treatment and a marked delay in DSB repair mechanisms. Furthermore, PA28γ rapidly accumulates at sites of DNA damage where it recruits proteasomes (20S but also 19S particles), whose proteolytic activity is required for an effective DNA damage response (DDR) to take place [143].

#### 3.2.10. Role in Cardiac Hypertrophy

By targeting for 20S degradation the protein phosphatase 2A catalytic subunit α (PP2Acα), PA28γ was recently reported to cause increased phosphorylation and nuclear export of forkhead box protein O (FoxO) 3a with a subsequent decrease in superoxide dismutase 2 (SOD2), accumulation of reactive oxygen species (ROS), and emergence of cardiac hypertrophy [144].

#### 3.2.11. Role in SARS-CoV-2 Infection

A recently published investigation would seem to indicate that PA28γ, similar to what previously reported for hepatitis C virus (HCV) core protein [145,146], is able to promote degradation of the SARS-CoV-2 nucleocapsid protein (nCoV N) by 20S proteasome [147]. In fact, intracellular levels of overexpressed nCoV N and PA28γ proteins were found to inversely correlate in 293 T cells, while in vitro translated nCoV N protein was degraded by purified 20S-PA28γ proteasomes. Although intriguing and potentially of extreme importance, these preliminary results, however, need to be further investigated and validated before a role of PA28γ in SARS-CoV-2 infection and in the etiopathogenesis of coronavirus disease 2019 (COVID-19) can be definitely established.

### 3.3. Biochemical Properties

#### 3.3.1. Stimulation of Peptide Hydrolysis

As previously mentioned, PA28s were originally identified due to their capability to markedly enhance hydrolysis by 20S proteasome of short (3–4 residues) fluorogenic peptides. Specifically, kinetic analyses have shown that PA28αβ increases the V_max_ and decreases the K_m_ for peptide hydrolysis by all three main proteasomal peptidase activities [58,60]. Subsequent biochemical studies confirmed that both the native and recombinant PA28α subunits are able to enhance degradation by 20S particles of several short fluorogenic substrates [41,43,148,149]. On the other hand, the situation regarding the ability of PA28γ to stimulate the different peptidase activities of the 20S proteasome appears to be more complex. In fact, PA28γ was initially described as able to exclusively activate the trypsin-like cleavage specificity of the 20S particle [43,59]. However, later analyses reported marked stimulation of the proteasomal chymotryptic and caspasic sites both in vitro [49,61,150] and in vivo [130], even in the absence of a purification step with ammonium sulfate, which was reported to expand the stimulatory properties of PA28γ [151]. Most importantly, the extent of 20S stimulation achieved by PA28γ differs between the three active sites and also varies for alternative fluorogenic peptides used to probe the same proteasome peptidase site [43,59,61,150]. Therefore, although there is little doubt that, as with other members of the PA28 family, PA28γ causes opening of the proteasomal gate, it is also evident that other complementary molecular mechanisms must contribute to precisely determine and regulate its stimulatory properties. In this regard, however, it still remains to be definitively established, as in the case of PA28αβ, whether it induces allosteric modifications of proteasomal catalytic sites and/or whether the charged or polar residues that almost completely line its longitudinal channel impose a constraint on the passage of specific peptides on the basis of their physical–chemical properties.

#### 3.3.2. Stimulation of Protein Breakdown

Of great interest, 15 years ago for the first time it was convincingly demonstrated that PA28γ was able to promote proteasomal degradation in vivo of a full-length oncogenic protein, the steroid receptor coactivator-3 (SRC-3AIB1), and in vitro by purified 20S particle in an ubiquitin- and ATP-independent proteolytic reaction [152]. This finding was unexpected, since until that moment PA28s had always been described as unable to enhance the proteolytic activities of 20S proteasomes, although hydrolysis of full-length proteins (e.g., casein, lysozyme, and serum albumin) was investigated only with regards to the role of PA28αβ [58,60]. Since then, several other proteins were identified whose degradation appears to depend on PA28γ in vivo and whose hydrolysis can be supported by PA28γ-activated 20S proteasomes in vitro [75]. Of note, most of these proteins, such as p16, p19, p21, c-Myc, GSK-3β, HCV core protein, and PTTG1, are involved in the control of molecular pathways related to cell growth and proliferation, apoptosis, and oncogenic transformation [99,113,124,153,154], which helps to account for the large variety of biological functions of PA28γ previously highlighted. Thus, although it is now widely accepted that PA28γ plays a central role in a proteolytic mechanism specifically involving the 20S particle, and which is distinct from the canonical ubiquitin- and ATP-dependent degradative pathway carried out by the 26S proteasome, it is still unknown how common this alternative degradative pathway is. Furthermore, the situation is even more complex since PA28γ has also been reported to indirectly promote degradation of the tumor suppressor P53 by enhancing its MDM2-mediated ubiquitination and subsequent ATP-dependent hydrolysis by the 26S proteasome [108]. Moreover, this stimulatory function on proteolysis seems to be specific for PA28γ, since a large body of evidence produced over the years clearly indicates that the highly similar homolog PA28αβ is unable to promote proteasomal degradation of any protein, even if ubiquitinated or completely unfolded [37,58,60]. Since the overall structures of PA28α, β, and γ monomers are highly conserved, this clear difference in their biochemical properties is likely to rely on the only divergent part between the three paralogs, the so-called homolog-specific inserts connecting helix 1 and 2, which are not resolved in crystal structures but which are very likely to protrude from the upper surface of the heptameric ring all around the pore of the longitudinal inner channel [31,40]. Interestingly, these loops are particularly extended in PA28γ, and based on this observation it would be tempting to speculate that they may be somehow involved in grasping unfolded substrates and channeling them towards the entrance pore of the activator.

## 4. Perspectives and Concluding Remarks

The specific molecular features that make a protein substrate susceptible to be degraded by PA28γ-20S particles have long gone unidentified. In this respect, it was observed that most of the full-length proteins whose degradation is enhanced by PA28γ share the common characteristic of being completely or in large part intrinsically unstructured [155,156,157]. However, it is generally believed that other specific characteristic, yet to be identified, in tridimensional structures and/or chemical-physical properties are responsible for their susceptibility to PA28γ-enhanced degradation [157]. In this respect, by assessing the rates of in vitro hydrolysis by the 20S proteasome of several naturally or chemically unfolded proteins, we recently demonstrated that PA28γ strongly accelerates the proteasomal degradation of all unstructured proteins tested, while it is completely inactive toward their native, folded counterparts [61]. Since this result was obtained with several unrelated proteins (in terms of size, chemical–physical properties, and cellular localization and functions), it strongly indicates that the lack of tightly folded three-dimensional structure is the major determinant for susceptibility of a polypeptide substrate to PA28γ pro-degradative stimulatory activity. Therefore, since several studies suggest that a considerable part of the eukaryotic proteome is constituted by proteins that lack a fixed or ordered three-dimensional structure [158], and whose degradation is at least partially ubiquitin-independent [159], it seems logical to hypothesize that PA28γ might play a role in the pathway of protein breakdown, especially in the nucleus, which is much more significant and general than previously imagined. In principle, this activating function of PA28γ on the proteolytic pathway could be even more essential under conditions of chemical or physical stress, which promote unfolding of native proteins that need to be quickly and efficiently degraded (if they cannot be refolded by the molecular chaperones network), before they misfold and aggregate, thus causing a serious threat to cell viability [160]. Further studies aimed at investigating the in vivo role of PA28γ under situations of stress challenge will be required to investigate this hypothesis and to identify its specific functions in the context of cellular adaptive responses that might have been lost, or highly underestimated, in previous analyses focusing on other physiological conditions.

Interestingly, recent phylogenetic studies show that PA28γ is most similar to the common ancestor of the PA28 activator family, and most likely retains its original functions, while PA28α and PA28β appeared later and evolved very rapidly to perform new tasks related to the interferon-γ inducible MHC class I system [81]. Moreover, biochemical characterization of a PA28γ homolog in *Dictyostelium discoideum* led to the conclusion that PA28γ-20S proteasomes could represent early unique nuclear proteases of eukaryotic cells [82]. Therefore, it seems likely that PA28γ has maintained the ancient property of promoting degradation of unstructured long polypeptides and proteins, while the phylogenetically more recent PA28α and PA28β lost the ability to stimulate protein hydrolysis, gaining a more pronounced capacity to modify the spectrum of peptides released by proteasomes in a functional way to favor MHC class I antigen presentation. Accordingly, by combining HP-size exclusion chromatography with LC-MS/MS tandem mass spectrometry sequencing and signal intensity-based relative quantification, it was demonstrated that PA28αβ profoundly modifies the entire spectrum of peptides generated by the 20S proteasome during degradation of full-length proteins, with a clear reduction of mean and median size of products, and a concomitant preferential release of specific, more hydrophilic, and longer peptides [37]. Conversely, no overall difference in terms of mean and median size was detected when a similar analysis was performed on the products released by the 20S proteasome in the presence or absence of PA28γ [61], although in related studies did demonstrate an effect on the rates of generation of specific individual peptides [131]. Overall, these data seem to confirm that PA28γ has a prevalent action aimed at increasing the degradation rates of unstructured proteins, while PA28αβ exerts a function mainly directed at modifying the spectrum of peptides released by the proteasome. Moreover, our data regarding PA28γ-enhanced 20S proteasome degradation of unfolded proteins emphasize the necessity to be extremely cautious when interpreting the results of in vitro degradation assays, especially when aiming to generalize molecular mechanisms to the actual situation in vivo. In fact, in vitro degradative experiments generally measure proteasomal hydrolysis of purified eukaryotic recombinant substrate proteins expressed in *E. coli* or, alternatively, translated in reticulocyte lysate systems. The correct three-dimensional folding of these substrates should, therefore, always be carefully assessed before it can be assumed that their in vitro hydrolysis by purified PA28γ-20S proteasomes represents a bona fide demonstration that degradation occurs in the same way in vivo.

In conclusion, much has been learned in the last 15 years about the biochemical properties and biological functions of PA28γ. New discoveries have been made at a rapid pace, especially in recent years, as shown by the ever-increasing number of biological processes in which PA28γ is reported to be involved and the ever-increasing quantity of protein substrates whose degradation is attributed to its specific activity. Despite this, many questions about its role and functions have still not been definitively answered and will therefore need to be addressed in the near future. Both in vitro and in vivo studies will be necessary to understand: (I) what proportion of the cellular proteome is subject to proteasomal degradation through the activity of PA28γ in an ATP- and ubiquitin- independent process; (II) whether this process in vivo concerns indiscriminately all intrinsically disordered proteins or only a part of them, and in this case what are the other molecular and cellular determinants of the susceptibility to degradation induced by PA28γ; (III) whether this degradative pathway exclusively concerns proteins localized in the nucleus or, at least in some phases of the cell cycle, may also involve cytoplasmic proteins; and (IV) what is the role of the degradation by PA28γ in specific cellular situations, such as the stress response or in pathological conditions in which there is an increased generation of unfolded/misfolded proteins that imposes a serious threat to cell viability. In this regard, it will also be important to investigate the functional interactions between PA28γ and molecular chaperones, which could act by protecting the temporarily unfolded proteins from the activity of PA28γ, but hypothetically also actively transfer them to the activator so that they can be degraded by the proteasome. The answers to all these important questions will hopefully benefit from the introduction of specific PA28γ inhibitors that may be developed in the next few years, and from specific crystallographic and electron microscopy structural data of PA28γ alone and in association with 20S proteasome that are currently missing.

## Figures and Tables

**Table 1:** Effects of interferon-γ on PA28γ expression.

mRNA Level	Protein Level	Ref.
unaffected	strongly reduced	[77]
not checked	strongly reduced (in mice liver) unaffected (in several cell lines)	[88]
slightly and transiently increased	not checked	[87]
slightly and transiently increased	not checked	[84]

## Data Availability

Not applicable.
